# Regulation of YKL-40 expression by corticosteroids: effect on pro-inflammatory macrophages in vitro and its modulation in COPD in vivo

**DOI:** 10.1186/s12931-015-0314-3

**Published:** 2015-12-22

**Authors:** L. I. Z. Kunz, E. F. A. van’t Wout, A. van Schadewijk, D. S. Postma, H. A. M. Kerstjens, P. J. Sterk, P. S. Hiemstra

**Affiliations:** Department of Pulmonology, Leiden University Medical Center, Albinusdreef 2, NL-2333 ZA Leiden, The Netherlands; Department of Pulmonology, University of Groningen, University Medical Center Groningen, Groningen, The Netherlands; Department of Respiratory Medicine, Academic Medical Center, Amsterdam, The Netherlands

## Abstract

**Background:**

Macrophages constitute a heterogeneous cell population with pro- (MΦ1) and anti-inflammatory (MΦ2) cells. The soluble chitinase-like-protein YKL-40 is expressed in macrophages and various other cell types, and has been linked to a variety of inflammatory diseases, including COPD. Dexamethasone strongly reduces YKL-40 expression in peripheral blood mononuclear cells (PBMC) in vitro. We hypothesized that: a) YKL-40 is differentially expressed by MΦ1 and MΦ2, b) is decreased by corticosteroids and c) that long-term treatment with inhaled corticosteroids (ICS) affects YKL-40 levels in serum and sputum of COPD patients.

**Methods:**

Monocytes of healthy subjects were cultured in vitro for 7 days with either GM-CSF or M-CSF (for MΦ1 and MΦ2, respectively) and stimulated for 24 h with LPS, TNFα, or oncostatin M (OSM). MΦ1 and MΦ2 differentiation was assessed by measuring secretion of IL-12p40 and IL-10, respectively. YKL-40 expression in macrophages was measured by quantitative RT-PCR (qPCR) and ELISA; serum and sputum YKL-40 levels were analyzed by ELISA.

**Results:**

Pro-inflammatory MΦ1 cells secreted significantly more YKL-40 than MΦ2, which was independent of stimulation with LPS, TNFα or OSM (*p* < 0.001) and confirmed by qPCR. Dexamethasone dose-dependently and significantly inhibited YKL-40 protein and mRNA levels in MΦ1. Serum YKL-40 levels of COPD patients were significantly higher than sputum YKL-40 levels but were not significantly changed by ICS treatment.

**Conclusions:**

YKL-40 secretion from MΦ1 cells is higher than from MΦ2 cells and is unaffected by further stimulation with pro-inflammatory agents. Furthermore, YKL-40 release from cultured monocyte-derived macrophages is inhibited by dexamethasone especially in MΦ1, but ICS treatment did not change YKL-40 serum and sputum levels in COPD. These results indicate that YKL-40 expression could be used as a marker for MΦ1 macrophages in vitro, but not for monitoring the effect of ICS in COPD.

**Trial registration:**

ClinicalTrials.gov, registration number: NCT00158847

## Background

YKL-40 (or chitinase 3-like-1 [CHI3L1], breast regression protein [BRP]-39 or human cartilage glycoprotein-39 [HC gp-39]) is a chitinase-like protein which is found in humans [1]. It is expressed by various cell types, including neutrophils and macrophages [2–4], while macrophages have been identified as its main cellular source [2, 5]. Monocytes do not express YKL-40, and YKL-40 expression appears to be associated with later stages of macrophage differentiation [2, 6]. Although its biological function is largely unknown, YKL-40 has been suggested to play a major role in a variety of processes, including epithelial-mesenchymal transition, migration and proliferation of (malignant) cells, angiogenesis, tissue remodeling and inflammation [7–9]. Furthermore, YKL-40 has been implicated in several acute and chronic inflammatory diseases, including asthma and COPD [10, 11]. Several studies have shown that YKL-40 levels are higher in sputum and serum of COPD patients compared to asymptomatic smokers [12, 13].

These data suggest that macrophages may also be a major source of YKL-40 in inflammatory lung diseases such as COPD, a disease in which macrophages play an important role [14]. It is however unknown whether YKL-40 expression is restricted to a subset of macrophages. This is a relevant question, since macrophages constitute heterogeneous cell populations with various functions. Pro-inflammatory, or classically activated macrophages (type I, MΦ1), show pro-inflammatory properties by secreting pro-inflammatory cytokines such as interleukin (IL)-12, have antigen presenting capacity and promote Th1 immunity. In contrast, anti-inflammatory, or alternatively activated macrophages (type II, MΦ2), demonstrate anti-inflammatory characteristics with expression and secretion of anti-inflammatory cytokines, such as IL-10 and promote development of regulatory T cells [15]. Furthermore, Di Rosa et al. observed higher expression of YKL-40 in monocyte-derived MΦ1 [16].

Dexamethasone strongly suppresses YKL-40 expression in cultured monocyte-derived macrophages [17]. In line with these findings, rheumatoid arthritis patients treated with methotrexate and prednisolone had lower serum YKL-40 concentrations compared to patients treated without prednisolone [18]. However, it is unknown if YKL-40 levels are influenced by treatment with inhaled corticosteroids (ICS) in COPD and could therefore be used to monitor treatment response. Furthermore, it is not known whether modulation by steroids is a direct genomic anti-inflammatory effect or due to e.g. epigenetic mechanisms [19–22].

Based on these observations we hypothesized that YKL-40 expression is directed by macrophage polarization, with a higher expression in monocyte-derived MΦ1 compared to MΦ2. Furthermore, we hypothesized that steroids decrease YKL-40 secretion and expression in MΦ1 and that YKL-40 in serum and sputum of COPD patients is decreased by treatment with ICS. The results from this study show that monocyte-derived MΦ1 secrete and express more YKL-40 than MΦ2. YKL-40 expression by cultured MΦ1 cells is steroid sensitive as its production is decreased by dexamethasone. Finally, YKL-40 levels in sputum and serum do not differ between COPD patients treated for 30 months with ICS or placebo.

## Methods

### Cell culture

Monocytes were isolated from buffy coats of healthy blood donors (Sanquin Blood Bank, Leiden, The Netherlands) using anti-CD14 microbeads (Miltenyi Biotec, Auburn, CA, USA) according to the manufacturer’s protocol. MΦ1 and MΦ2 were derived as described previously [23, 24]. Briefly, cells were cultured for six days in RPMI 1640 medium in 48 well plates (Invitrogen, Life Technologies, Bleiswijk, The Netherlands) containing 10 % fetal calf serum (FCS, Invitrogen), 2 mM L-glutamine, 100U/ml penicillin and 100 μg/ml streptomycin (all Bio Whittaker, Walkersville, MD, USA) at 37 °C in 5 % CO_2_ atmosphere with either GM-CSF (5 ng/ml, Invitrogen) or M-CSF (50 ng/ml, R&D Systems, Minneapolis, MN, USA) to obtain MΦ1 and MΦ2, respectively [13, 14]. Differentiated macrophages were stimulated with the pro-inflammatory stimuli lipopolysaccharide (LPS, from *Pseudomonas aeruginosa*, 100 ng/ml, Sigma-Aldrich, St. Louis, MO, USA), TNF-α (10 ng/ml, Peprotech, Rocky Hill, NJ, USA) or oncostatin M (OSM, 100 ng/ml, R&D Systems) for 24 h. Dexamethasone (0.1, 0.3 and 1nM, Sigma) was added during differentiation at day 0, 3 and/or day 7. The demethylating agent 5-AZA-2’-deoxycytidine (5-AZA, 0.1, 1 and 10 μM, Sigma) was added during differentiation. Every day 100 μl per well was removed and replaced by fresh medium containing growth factors and 5-AZA.

### GLUCOLD study

Serum and sputum supernatants were obtained from patients with moderate to severe COPD who participated in the GLUCOLD (Groningen and Leiden Universities Corticosteroids in Obstructive Lung Disease) study [25]. Patients were steroid-naive at baseline and were subsequently randomized to one of four inhaled treatments, all twice daily: 6- or 30-month fluticasone propionate dry-powder inhaler (500 μg, group 1 and 2, respectively), 30-month fluticasone with salmeterol (500/50 μg, group 3) or 30-month placebo (group 4). In this mechanistic study, we only used data from the compliant patients (≥70 % of the prescribed dose of treatment) of groups 2 and 3 combined to increase power, and group 4. At baseline and after 30 months of treatment, serum and sputum samples were collected. Sputum induction and processing were performed as previously described [26, 27]. Cell free supernatants of serum and sputum were stored at −80 °C. The ethics committees of Leiden University Medical Center and University Medical Center Groningen approved the study and all patients provided written informed consent.

### Enzyme-linked Immunosorbent Assay (ELISA)

**Fig. 1:**
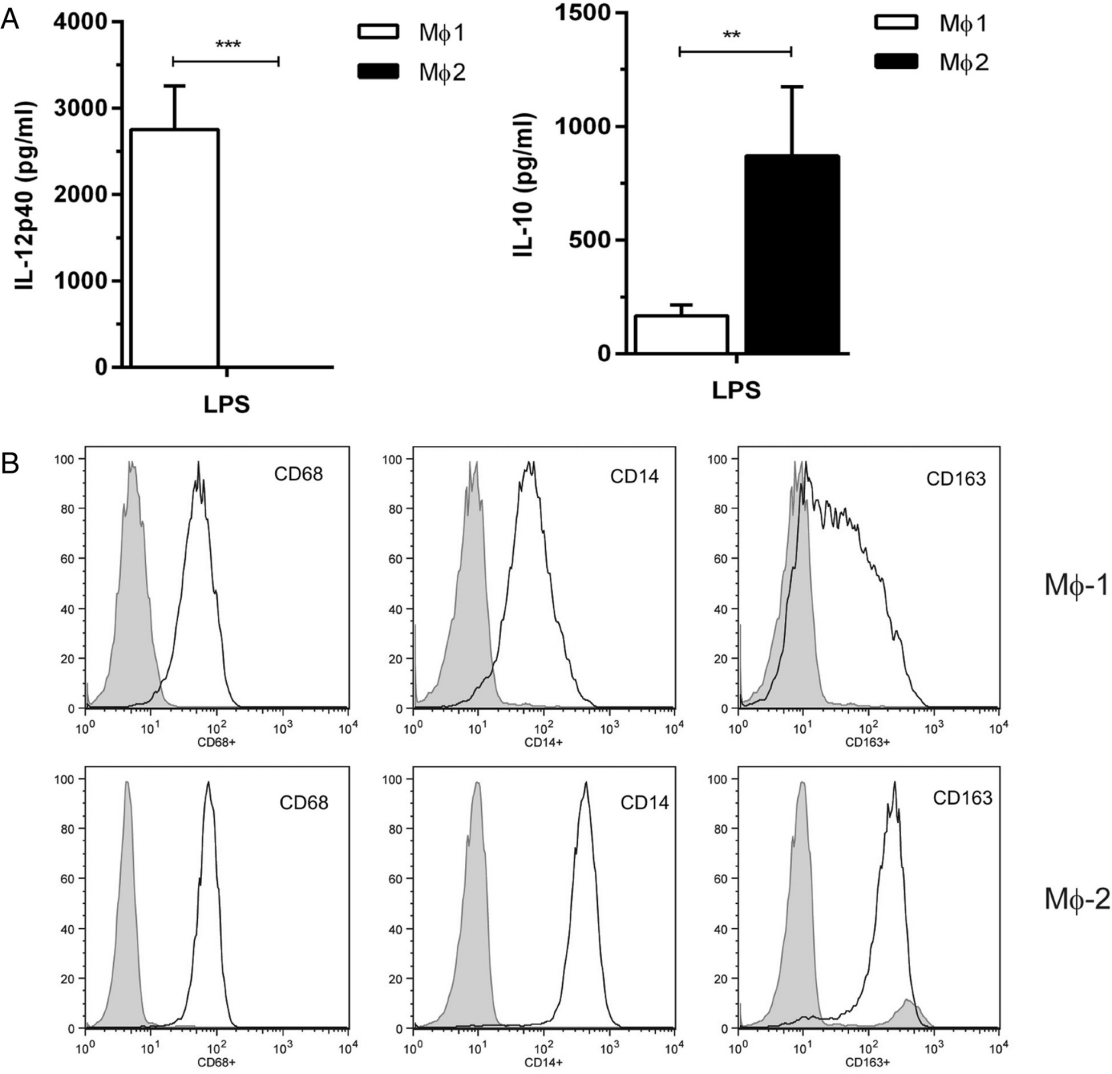
Characterization of differentiated MΦ1 and MΦ2. **a**: IL-12p40 (left panel) and IL-10 (right panel) in supernatants of MΦ1 and MΦ2, respectively, stimulated with LPS for 24 h. Data represent means with SEM of 6 donors, ** *p* < 0.01 or *** *p* < 0.001 between MΦ1 and MΦ2. **b**: Flow cytometry analysis for CD68, CD14 and CD163 (left, middle and right panel, respectively) for MΦ1 and MΦ2 (upper and lower panel, respectively)

Commercially available ELISA kits were used to detect IL-12p40 (IL-12/IL-23p40, R&D Systems; sensitivity 62.5 pg/ml) and IL-10 (Sanquin, Amsterdam, The Netherlands; sensitivity 4.1 pg/ml), to confirm that the monocytes were adequately differentiated towards MΦ1 and MΦ2, respectively (Fig. 1a). YKL-40 ELISA (R&D Systems; sensitivity 16 pg/ml [10]) was performed on cell culture supernatant, serum and sputum supernatant. The absorbance was measured at 450 nm using a Microplate reader (model 680; Bio-Rad, Hercules, CA, USA) and Microplate Manager software (version 5.2.1, Bio-Rad).

### Flow cytometry

Cell surface markers were assessed by standard flow cytometry using a FACS Calibur cytometer (Becton and Dickinson, La Jolla, CA, USA) and CellQuest Pro software after staining with specific APC-conjugated CD163 and CD14 (both BD Biosciences/Pharmingen (Temse, Belgium), FITC-conjugated CD68 (eBioscience, Vienna, Austria) and/or goat-anti-human YKL-40 (R&D systems) detected by Alexa Fluor 594 F(ab’)_2_ fragment of goat anti-mouse (Invitrogen) as secondary antibody. Cells were incubated with the antibodies for 30 min on ice in PBS containing 0.5 % BSA (w/v) and 0.2 % sodium azide (w/v) (both Sigma). After fixation with Cytofix/Cytoperm buffer, intracellular staining was performed in Perm/Wash buffer (both BD Biosciences). Flow cytometric analysis confirmed that the differentiated monocytes were CD14^+^ and CD68^+^, and that the majority of MΦ2 were CD163^+^ (Fig. 1b). Therefore, we concluded that the monocytes were properly differentiated into MΦ1 and MΦ2 cells.

### Immunofluorescence staining

YKL-40 and CD68 expression in sputum cytospins was demonstrated using immunofluorescence. Cytospins were fixed in 4 % formaldehyde in PBS for 30 min, followed by antigen retrieval in citrate solution pH6.0 (DAKO, Glostrup, Denmark) for 30 min and cooled on ice. The primary antibodies goat-anti-human YKL-40 (R&D systems, dilution 1:25) and mouse-anti-human CD68 (clone PG-M1, DAKO, dilution 1:50), diluted in PBS/1 % BSA (w/v) were incubated together overnight. Alexa Fluor568 donkey-anti-goat and Alexa Fluor 488 donkey-anti-mouse (Invitrogen, Eugene, Oregon, USA, dilution of both 1:200) were incubated in a dark environment for 30 min. The cytospins were covered with Vectashield with DAPI (Vector Laboratories, Inc. Burlingame, CA, USA). Photographs were taken with a confocal microscope.

### Quantitative reverse-transcriptase polymerase chain reaction (qPCR)

RNA was isolated using Qiagen RNeasy mini kit (Qiagen, Venlo, The Netherlands) and cDNA was synthesized in equal amounts per experiment. Quantitative reverse-transcriptase polymerase chain reaction (qPCR) was performed with the primers for YKL-40 and LL-37 as presented in Table 1. QPCR was performed on the iCycler PCR device using iQ SYBR Green Supermix (Bio-Rad) for 40 cycles at 58 °C. Relative mRNA concentrations of ACTB and ATP5B (GeNorm, PrimerDesign Ltd., Southampton, UK) were used as housekeeping genes for human genes.

**Table 1:** Primer pairs used for quantitative reverse-transcriptase polymerase chain reaction (qPCR)

	Forward	Reverse
YKL-40 (CHI3L1) (154 base pairs)	CTG TGG GGA TAG TGA GGC AT	CTT GCC AAA ATG GTG TCC TT
LL-37 (CAMP) (249 base pairs)	TCA TTG CCC AGG TCC TCA G	TCC CCA TAC ACC GCT TCA C

### Statistical analysis

Differences within one cell type and between cell types were analyzed by one-way and two-way ANOVA, respectively. Paired and unpaired tests were used for evaluating YKL-40 levels in serum and sputum within and between treatments, respectively, using only data from subjects with availability of samples from both baseline and 30 months. Statistical analysis was performed with SPSS 22.0 software (SPSS Inc., Chicago, IL). Data are presented as means with standard error of the mean (SEM) for in vitro experiments and means with standard deviations (SD) for serum and sputum samples. Differences at p-values ≤0.05 were inferred as statistically significant.

## Results

### MΦ1 produce more YKL-40 compared to MΦ2

**Fig. 2:**
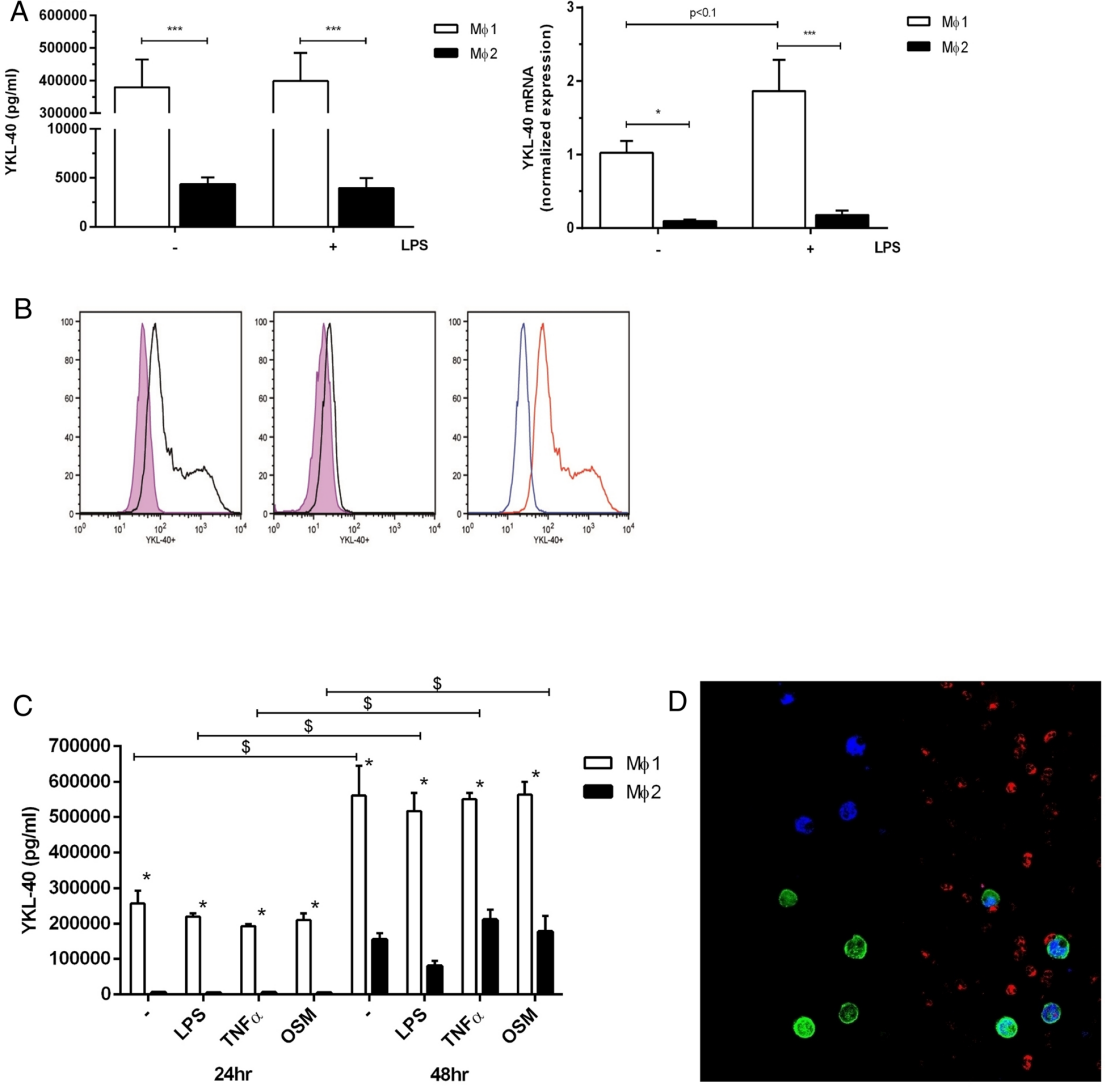
MΦ1 produce more YKL-40 compared to MΦ2. **a**: YKL-40 secretion measured by ELISA (left panel, 6 donors) and mRNA levels (normalized expression, right panel, 5 donors) in MΦ1 and MΦ2 after medium and LPS stimulation for 24 h. * *p* < 0.05 or *** *p* < 0.001 between MΦ1 and MΦ2. **b**: Flow cytometry analysis of MΦ1 and MΦ2 with and without anti-YKL-40 and secondary antibody. Left panel: MΦ1 with secondary antibody (purple) and MΦ1 with anti-YKL-40 and secondary antibody (black); middle panel: MΦ2 with secondary antibody alone (purple) and MΦ2 with anti-YKL-40 and secondary antibody (black); right panel: MΦ1 and anti-YKL-40 with secondary antibody (red) and MΦ2 and anti-YKL-40 with secondary antibody (blue). **c**: YKL-40 secretion in MΦ1 and MΦ2 after 24 and 48 h of stimulation with medium, LPS, TNFα and oncostatin M (OSM). Data represent mean and SEM of 4 and 3 donors (24 and 48 h, respectively). *: *p* < 0.001 between MΦ1 and MΦ2 for corresponding stimulus and time. $: *p* < 0.001 between corresponding stimulus at different time points. **d**: Immunofluorescence staining on sputum cytospin of a COPD patient (blue: DAPI; red: YKL-40; green: CD68)

Secretion of YKL-40 by MΦ1 was markedly higher compared to MΦ2 (*p* < 0.001) and not further increased by stimulation for 24 h with 100 ng/ml LPS compared to medium (*p* < 0.001; Fig. 2a, left panel). qPCR confirmed these results, i.e. MΦ1 express more YKL-40 than MΦ2 with and without LPS stimulation (10-fold higher in MΦ1 vs MΦ2, *p* < 0.001; Fig. 2a, right panel), with a trend towards more YKL-40 expression in LPS-stimulated MΦ1 (*p* < 0.1). Flow cytometry analysis also showed that MΦ1 cells express more YKL-40 compared to MΦ2 (Fig. 2b). In addition, MΦ1 secreted more YKL-40 compared to MΦ2 which was irrespective of further stimulation with TNF-α, and OSM for 24 or 48 h, respectively (all *p* < 0.001, Fig. 2c). Immunofluorescence staining showed that only a minority of macrophages in sputum of COPD patients was positive for YKL-40 as shown by co-staining with CD68 (Fig. 2d).

### YKL-40 expression is inhibited by dexamethasone

**Fig. 3:**
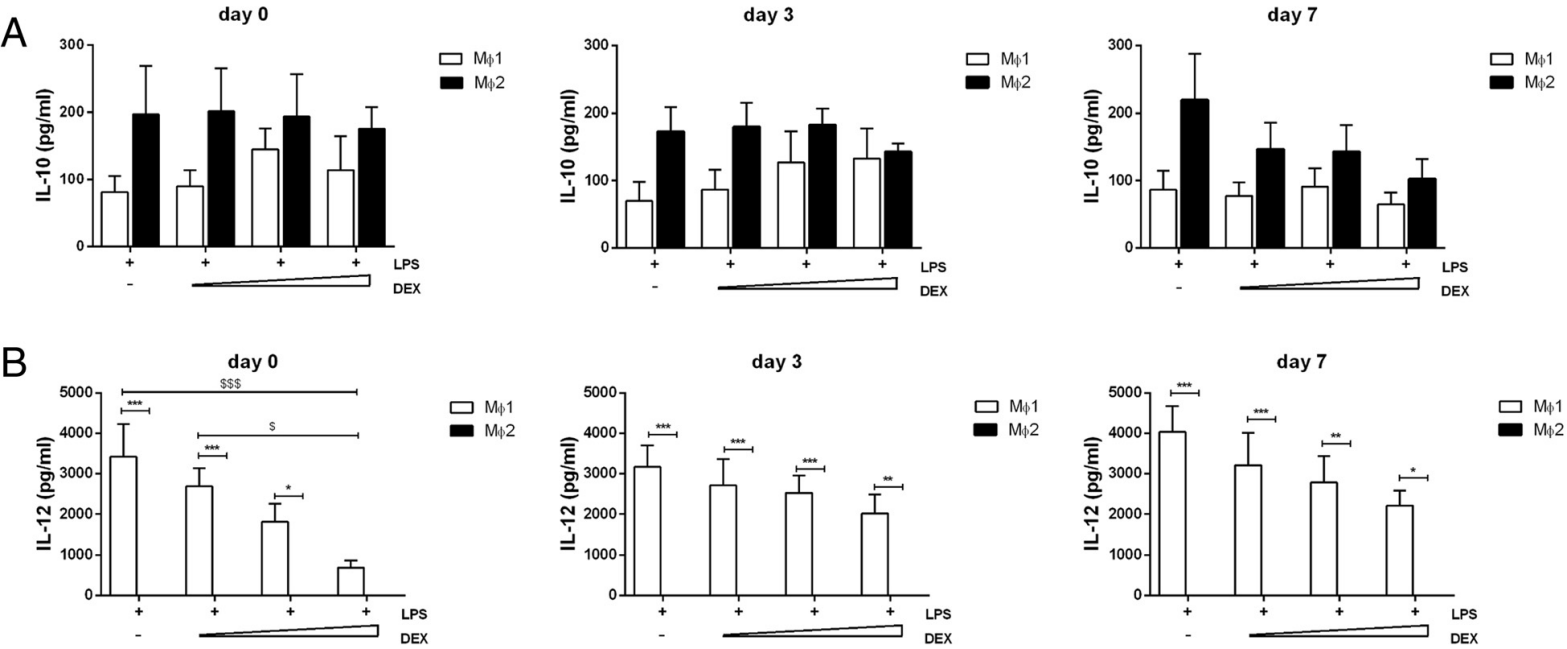
IL-12p40 secretion is dose-dependently inhibited by dexamethasone when added on day 0. IL-10 and IL-12p40 secretion with addition of dexamethasone at day 0, 3 and 7 of differentiation (0.1, 0.3 and 1nM) (**a** and **b**, left, middle and right panel, respectively). Data represent means and SEM (*n* = 3 donors). * *p* ≤ 0.05, ** *p* < 0.01 or *** *p* < 0.001: between MΦ1 to MΦ2 with corresponding stimulus. $ *p* ≤ 0.05 or $ *p* < 0.001 between MΦ1 at different concentrations of dexamethasone

**Fig. 4:**
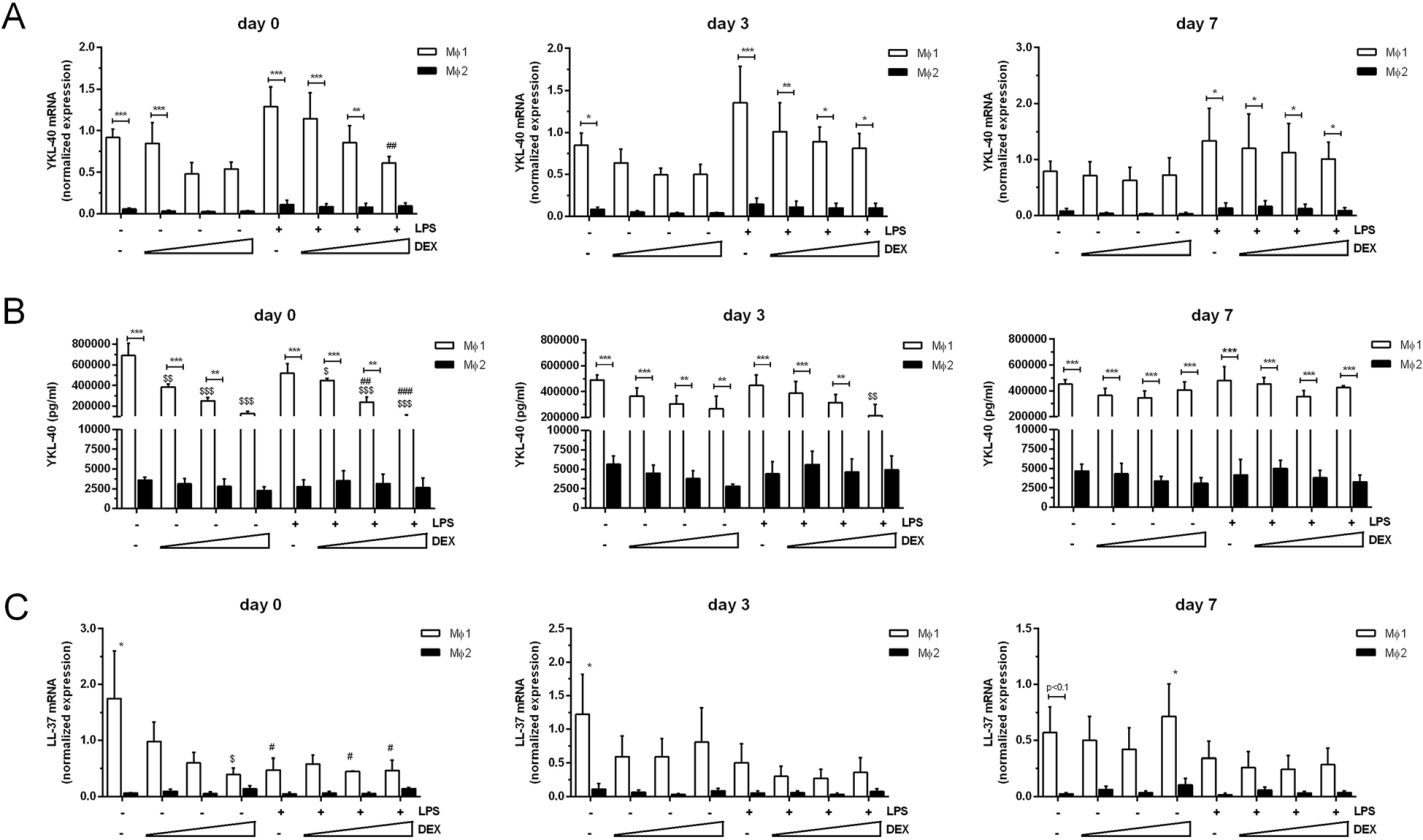
YKL-40 expression and protein secretion is inhibited by dexamethasone. YKL-40 mRNA levels (normalized expression) and YKL-40 protein secretion (**a** and **b**, respectively) in MΦ1 and MΦ2 with addition of dexamethasone at day 0, 3 and 7 of differentiation (0.1, 0.3 and 1nM) (left, middle and right panel, respectively). LL-37 mRNA levels (normalized expression) after 0, 3 and 7 days of differentiation with dexamethasone (0.1, 0.3 and 1nM) (**c** left, middle and right panel, respectively). Data represent means and SEM (*n* = 3 donors). * *p* ≤ 0.05, ** *p* < 0.01 or *** *p* < 0.001 compared to MΦ2 with corresponding stimulus. $ = *p* ≤ 0.05, $$ *p* < 0.01 or $$$ *p* < 0.001 compared to corresponding unstimulated cells. ##: *p* < 0.01 or ### *p* < 0.001 compared to corresponding LPS stimulated cells

To investigate the effect of steroids on macrophage YKL-40 expression, we first assessed its effect on expression of the MΦ1 and MΦ2 markers IL-12p40 and IL-10. Whereas dexamethasone did not affect IL-10 secretion, it inhibited IL-12p40 secretion, especially when added at day 0 (Fig. 3a and b, respectively). In line with these findings, dexamethasone also dose-dependently inhibited YKL-40 expression and secretion mainly in MΦ1 (Fig. 4a and b, respectively). Again this effect was most pronounced when dexamethasone was added from the start of the differentiation at day 0 (Fig. 4a and b, left panel). Adding dexamethasone at later time points (day 3 or day 7) showed a trend for lower YKL-40 expression and secretion (Fig. 4a and b, middle and right panel, respectively), but failed to reach significance.

We have previously demonstrated that the human cathelicidin antimicrobial peptide hCAP/LL-37 directs macrophage differentiation towards MΦ1 cells [28]. In the course of the latter studies we also noted that hCAP18/LL-37 is preferentially expressed in MΦ1 cells (unpublished results). In the present study, we therefore used LL-37 expression for a comparison with YKL-40 expression. In line with the findings for IL-12p40 and YKL-40, the expression of LL-37 was also dose-dependently reduced by increasing doses of dexamethasone when added on day 0, but not on day 3 or day 7 (Fig. 4c).

Collectively these data indicate that dexamethasone inhibits YKL-40 expression, but that this effect is most likely explained by a more generic effect on MΦ1 development and is not selective for YKL-40 expression.

### YKL-40 expression is not affected by the demethylating agent 5-AZA

**Fig. 5:**
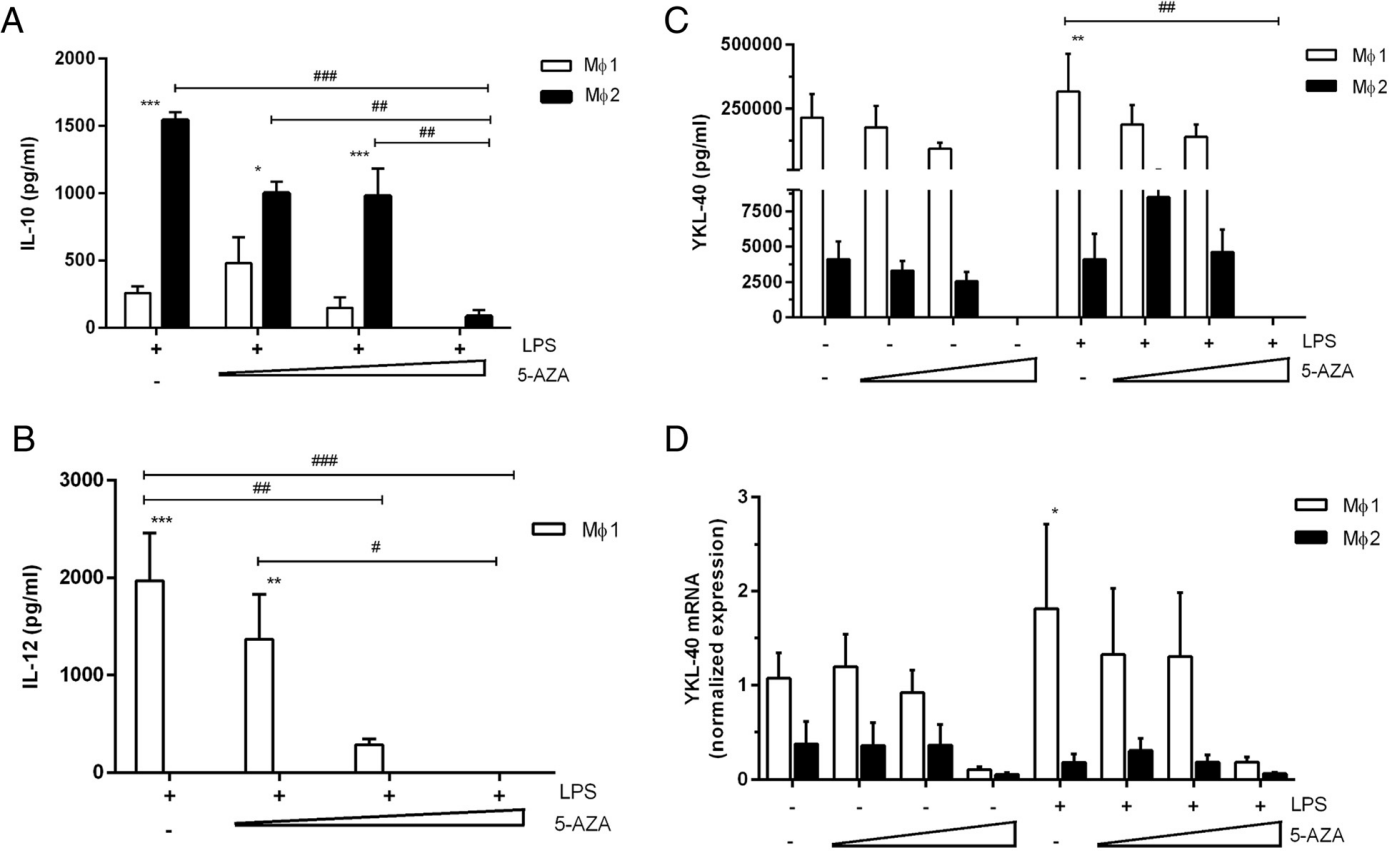
YKL-40 protein secretion and mRNA expression is inhibited by the demethylating agent 5AZA. IL-10 and IL-12p40 secretion after stimulation with 5-AZA (concentration 0.1, 1 and 10 μM) (**a** and **b**, respectively). YKL-40 protein secretion and mRNA expression in MΦ1 and MΦ2 after culturing with 5-AZA (concentration 0.1, 0.3 and 1nM) (**c** and **d**, respectively). Differentiated cells are stimulated with 100 ng/ml LPS for 24 h. Data represent mean and SEM (*n* = 3 donors). * *p* ≤ 0.05, ** *p* < 0.01 or *** *p* < 0.001 compared to MΦ2 with corresponding stimulus. # *p* ≤ 0.05, ##: *p* < 0.01 or ### *p* < 0.001 compared to corresponding LPS stimulated cells

Epigenetic mechanisms may contribute to CHI3L1 expression. This is supported by the finding of a single-nucleotide polymorphism (SNP) localized near the CpG island in the promoter region of the *CHI3L1* gene, which is associated with YKL-40 expression [19, 20]. To evaluate whether YKL-40 expression in monocyte-derived macrophages is influenced by the methylation status of the *CHI3L1* gene, macrophages were generated in the presence of the demethylating agent 5-AZA. We first observed that 5-AZA inhibited IL-12p40 secretion, and to a smaller extent also IL-10 secretion (Fig. 5a and b, respectively). In contrast, YKL-40 protein secretion and expression was not significantly affected by 5-AZA, although it needs to be noted that the highest concentration resulted in cell toxicity (Fig. 5c and d, respectively).

### YKL-40 levels in serum and sputum of COPD patients are not changed by treatment with inhaled corticosteroids

**Fig. 6:**
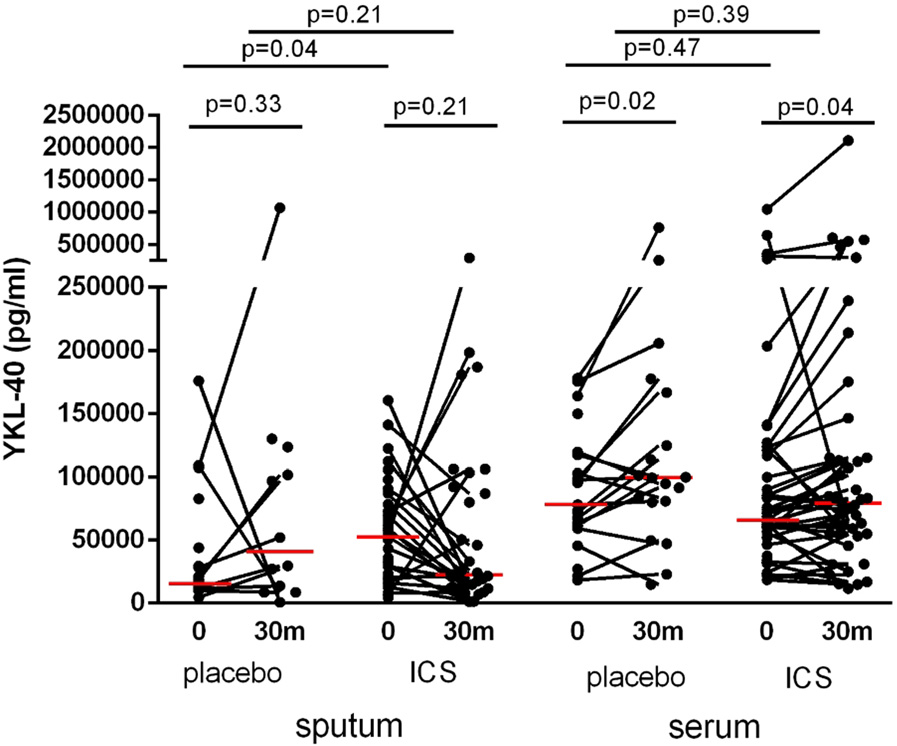
Sputum and serum YKL-40 protein levels of COPD patients before and after ICS treatment. YKL-40 levels in sputum and serum at baseline (0) and after 30 months (30 m) of inhaled corticosteroids (ICS) and placebo. For comparison of the levels between baseline and 30 months, we only included patients from whom samples were available at both time points (paired data). Each dot represent a single patient, red horizontal bars represent medians

We next used samples of the GLUCOLD study to investigate the effects of ICS treatment on serum and sputum YKL-40 levels in COPD patients. Baseline characteristics between the group of moderate-to-severe COPD patients treated with ICS and placebo were not significantly different as shown in Table 2. Of the 75 compliant patients, 70 serum samples and 59 induced sputum samples at baseline were available and suitable for analysis. Serum YKL-40 levels at baseline were significantly higher compared to sputum levels at baseline (respectively median 71 ng/ml versus 29 ng/ml, *p* < 0.001). ICS treatment did not significantly change YKL-40 levels in serum and sputum compared to placebo (both *p* > 0.05; Fig. 6).

**Table 2:** Patient characteristics of compliant patients at baseline of the GLUCOLD study

	ICS (*n* = 51)	Placebo (*n* = 24)
Gender (M/F) (n)	45/6	20/4
Age (yr)	61.7 (7.8)	59.4 (8.1)
Smoking (y/n) (n)	33/18	17/7
Packyears (yr)	48 (31–56)	42 (34–54)
Post-bronchodilator FEV_1_ (% pred)	62.5 (9.2)	61.2 (8.3)
Post-bronchodilator FEV_1_ (L)	2.02 (0.41)	2.00 (0.55)
Post-bronchodilator FEV_1_/IVC (%)	47.3 (8.8)	46.7 (9.0)
Serum YKL-40 (ng/ml)	66 (49–119)	78 (60–118)
Sputum YKL-40 (ng/ml)	52 (20–79)	18 (12–40)

The ICS group is a combination of the original 30-month fluticasone and 30-month fluticasone with salmeterol groups

Data represent mean with SD, median with interquartile range or numbers

*GLUCOLD* Groningen and Leiden Universities Corticosteroids in Obstructive Lung Disease, *ICS* inhaled corticosteroids, *FEV*_*1*_ forced expiratory volume in 1 s, *IVC* inspiratory vital capacity, *Pred* predicted

## Discussion

This study shows that secretion and expression of YKL-40, a chitinase-like protein, is higher in in vitro generated monocyte-derived MΦ1 than in MΦ2, and that YKL-40 expression is not further increased upon stimulation with several pro-inflammatory stimuli. In addition, YKL-40 release in vitro is strongly inhibited by dexamethasone especially in MΦ1, most likely due to an effect on differentiation. Addition of the demethylating agent 5-AZA did not significantly decrease YKL-40 release, but did decrease IL-12p40 production by MΦ1 and to a smaller extent IL-10 production by MΦ2 cells. YKL-40 levels in serum were significantly higher in serum than in sputum of COPD patients. Treatment of these patients for 2.5 years with inhaled corticosteroids did not significantly change serum and sputum YKL-40 levels compared to placebo. These results suggest that YKL-40 is a promising pro-inflammatory marker in in vitro cultured pro-inflammatory macrophages, but is less suitable for monitoring in vivo effect of treatment with steroids on YKL-40 in serum and sputum of COPD patients.

We show that YKL-40 is a novel marker of in vitro cultured monocyte-derived MΦ1, which is independent of LPS, OSM and TNFα. This is an important observation, since many established MΦ1 markers require additional stimulation to induce expression. Our data confirm and extend previous results [16, 17, 29], reporting higher CHI3L1 expression in classically activated macrophages compared to alternatively activated macrophages. In the latter studies, in contrast to our study, interferon-gamma (IFN-γ) and IL-4 were used for MΦ1 and MΦ2 polarization, respectively [16, 17]. We extended these data by differentiating monocytes with GM-CSF and M-CSF into MΦ1 and MΦ2, respectively, and explored the effect of further stimulation after differentiation with several pro-inflammatory stimuli. We found that dexamethasone efficiently suppressed YKL-40 expression and secretion in MΦ1, but that this was mainly explained by an inhibitory effect of dexamethasone on MΦ1differentiation, thus extending previous results [17].

We found that YKL-40 levels in serum were higher than in sputum of COPD patients. Serum YKL-40 levels of our group of patients were comparable with previous studies [13, 30, 31]. However, sputum YKL-40 levels with sputum processed using the whole sample method, were considerably lower than in studies using the selected plug method [12]. This is most likely due to dilution which is inherent to the whole sample method. After long-term treatment with ICS, we did not detect a significant change in YKL-40 levels in serum and sputum compared to placebo. To our knowledge, this is the first study that evaluates the long-term effect of ICS in serum and sputum YKL-40 levels of COPD patients. Therefore, this study presents new in vitro and in vivo data that may help to provide insight in the function of YKL-40.

The mechanisms that regulate YKL-40 expression in health and disease are partly understood. It has been demonstrated that YKL-40 expression is absent in monocytes, and markedly induced during macrophage differentiation, especially during the later stages of differentiation [2]. Promotor analysis of the *CHI3L1* gene revealed that especially Sp1, an ubiquitous transcription factor, is important for *CHI3L1* gene expression [6]. Possibly epigenetic mechanisms also contribute to *CHI3L1* expression. This is supported by the finding of a SNP localized near the CpG island in the promoter region of the *CHI3L1* gene, which is associated with YKL-40 expression [19, 20, 32]. Furthermore, hypomethylation of the *CHI3L1* gene in rheumatoid arthritis is associated with increased expression of YKL-40 [33, 34]. Therefore, *CHI3L1* gene expression may be regulated by transcription factors such as Sp1 and by DNA methylation status. Our observations with 5-AZA treatment do not support a role for DNA methylation in the expression of YKL-40 in MΦ1. Further studies into methylation status of the promotor of YKL-40, the role of histone modification and microRNAs are needed to define a role of epigenetic mechanisms in the expression of YKL-40 in (lung) macrophages.

The strength of our study is that it describes a thorough evaluation of a novel, potential pro-inflammatory macrophage marker using both in vitro and in vivo approaches. Well-characterized patients with COPD used long-term, randomized, placebo-controlled treatment with ICS. However, we were unable to detect an effect of a randomized treatment with ICS on YKL-40 serum and sputum levels. Nevertheless, our study has some limitations. First, we used in vitro cultured monocyte-derived macrophages from whole blood of healthy subjects that were differentiated towards MΦ1 and MΦ2 instead of lung-derived (e.g. alveolar) macrophages that were differentiated under the influence of the local environment. Since the culture systems do not fully reflect in vitro differentiation of macrophage subsets [35, 36], it needs to be noted that the effect of steroids on lung macrophages may differ from that on in vitro differentiated macrophages. Furthermore, in vivo a heterogeneous and intermediate macrophage population exists [37], which complicates the comparison with in vitro generated MΦ subsets. We therefore cannot formally exclude the possibility that this has contributed to our inability to detect an effect of inhaled corticosteroids on serum and sputum YKL-40 levels. Second, the demethylating agent 5-AZA demonstrated cell toxicity which might have influenced our results. However, we found a dose-dependent inhibition of YKL-40 expression and secretion, suggesting that DNA methylation status may contribute to regulation of YKL-40 expression in MΦ1, which is in line with studies posing that methylation of a part of the CpG island of the *CHI3L1* gene is associated with YKL-40 levels [19, 32].

How can we explain that serum and sputum YKL-40 levels of COPD patients were not significantly changed after long-term ICS treatment compared to placebo? This is unexpected since serum YKL-40 levels of rheumatoid arthritis patients rapidly decreased after one week of prednisolone [18]. However, the amount of inhaled fluticasone that reaches the systemic circulation is low [38], which could explain why serum YKL-40 levels did not significantly change with ICS therapy. In addition, lung macrophages in COPD have reduced glucocorticoid sensitivity [39, 40].

## Conclusions

YKL-40 is mainly expressed and secreted by MΦ1 and is not further increased by pro-inflammatory stimuli. YKL-40 release is inhibited by dexamethasone in MΦ1 in vitro, whereas long-term treatment of COPD patients with inhaled corticosteroids did not significantly change YKL-40 levels in serum and sputum. This suggests that YKL-40 is a potential marker for in vitro cultured pro-inflammatory macrophages and is not a valuable biomarker in serum and sputum of patients with COPD treated with inhaled corticosteroids.

## Abbreviations

5-AZA: 5-AZA-2’-deoxycytidine
BRP-39: Breast regression protein-39
BSA: Bovine serum albumin
COPD: Chronic obstructive pulmonary disease
ELISA: Enzyme-linked immunosorbent assay
FCS: Fetal calf serum
FEV_1_: Forced Expiratory Volume in 1 second
GLUCOLD: Groningen and Leiden Universities Corticosteroids in Obstructive Lung Disease
hCAP: Human cathelicidin antimicrobial peptide
HC gp-39: human cartilage glycoprotein-39
ICS: Inhaled corticosteroids
IL: Interleukin
LPS: Lipopolysaccharide
OSM: Oncostatin M
SNP: Single-nucleotide polymorphism
TNFα: Tumour Necrosis Factor-alpha
YKL-40: Chitinase 3-like-1
